# Complementary and alternative medicine in dermatomyositis: A scoping review

**DOI:** 10.1016/j.jdin.2025.06.001

**Published:** 2025-06-27

**Authors:** Ghizlane Yaakoubi, Maha Kazmi, Sarah Patterson, Anna Haemel

**Affiliations:** a Faculty of Medicine, Mohammed First University, Oujda, Morocco; b Department of Dermatology, University of California, San Francisco, San Francisco, California; c School of Medicine, University of California, Davis, Sacramento, California; d Department of Rheumatology, University of California, San Francisco, San Francisco, California

**Keywords:** alternative medicine, complementary medicine, dermatomyositis, idiopathic inflammatory myopathy, integrative medicine, myositis

*To the Editor:* Dermatomyositis (DM) is a rare autoimmune condition with characteristic skin changes, muscle inflammation, and potential multi-organ involvement.^1^ The current standard of care includes a combination therapy based on disease severity but adverse effects and variable efficacy prompt interest in complementary and alternative medicine (CAM). However, the safety, efficacy, and risk of CAM therapies among patients with DM remain inadequately understood.^1^ Through this scoping review, we aim to investigate existing literature on the use of CAM therapies among adult patients with DM.

Our scoping review followed the Preferred Reporting Items for Systematic Reviews and Preferred Reporting Items for Systematic Reviews and Meta-analyses (PRISMA) guidelines, utilizing PubMed, Embase, Web of Science, Google Scholar, and UC Library until March 2024. Articles were identified using search strings from these databases (Supplementary Methods, available via Mendeley at https://data.mendeley.com/datasets/cnwxbw8x48/1/files/dab4b5e7-f98c-4ded-8149-1e4d1a8f08f2). Two authors independently extracted the data (G.Y and M.K.), with conflicts resolved by consensus.

Seventeen articles were included (Supplementary Diagram, available via Mendeley at https://data.mendeley.com/datasets/43b6p5czjr/1). CAM therapies were categorized as (A) Beneficial and (B) Harmful, the latter associated with adverse outcomes, disease development, or exacerbation (Table I; Supplementary Tables, available via Mendeley at https://data.mendeley.com/datasets/49c3z8sg9z/1 and https://data.mendeley.com/datasets/n8x59pmjpt/1). Beneficial CAM studies described decreased mortality rates with Herbal Traditional Chinese Medicine (hTCM) interventions (31.64 vs 115.29 per 1000 person-years, *P* < .001), and improved quality of life (QoL) with creatine supplements (RR = 4.51; 95% CI: 2.33-8.74). Exercise interventions were linked to improvements of muscle strength of between 37% and 46% (*P* < .05), while mind-body practices (meditation, yoga) suggested modest overall strength improvements.^2^^,^^3^ In contrast, harmful CAM studies implicated a significant temporal association between Spirulina use and DM onset (*P* = .009), with related supplements containing Spirulina platensis, Aphanizomenon, and alfalfa showing similar patterns.^4^^,^^5^ Atypical therapies, including dessicated rattlesnake and Kava-Kava, were linked to development of bone necrosis and worsening myalgias, respectively. However, most studies were limited by small sample sizes and lack of controls.

**Table I:** Complementary and alternative medicine in dermatomyositis: summary of findings

CAM therapy	*N* of studies and type	Total patients	Key findings	Main limitations
A. Beneficial CAM therapies				
Herbal traditional Chinese medicine	1 (Cohort)	484	Decreased mortality (115.39 vs 31.64 per 1000 person-years (*P* < .001))	Did not specify DM from other IIM
Creatine supplements	1 (Systematic Review)	364	Improved quality of life (RR = 4.51; 95% CI: 2.33-8.74) and strength (8.47% increase; 95% CI 3.55-13.38)	Did not specify DM from other IIM
Mind-body medicine	3 (1 Cohort, 2 Case Reports)	14	Modest strength improvements; reduced pain and rash	Small sample size; lack of controls
Exercise therapies	2 (1 Cohort, 1 Review)	21	Improved muscle strength (*P* < .05)	Small sample size
Water-based therapies	1 (Case Report)	1	decreased pain and improved skin manifestations	Small sample size; lack of controls
Combined nutritional and mind-body therapies	2 (Case Reports)	4	Decreased pain; Improved strength; Decreased CPK levels	Small sample size; lack of controls
Other CAM therapies (HBO_3_)	1 (Case Report)	1	Improved QoL; decreased fatigue; decreased pain	Small sample size; lack of controls
B. Harmful CAM therapies				
Microalgae products (Spirulina, Chlorella), Echinacea, and Alfalfa	4 (2 Cohorts, 1 Review, 1 Case series)	825	Temporal association between use and DM onset/flare (*P* = .009), triggered disease exacerbation in previously controlled DM case	Incomplete outcome reporting
Other supplements (Rattlesnake meat powder, Kava-Kava)	2 (1 Case Report, 1 Case series)	12	Development of DM-like disease exacerbation, and other complications.	Small sample size; Lack of controls

*CAM*, Complementary and alternative medicine; *CPK*, creatine phosphokinase; *DM*, dermatomyositis; *HBO*_*3*_, hyperbaric ozone; *IIM*, idiopathic inflammatory myopathies; *QoL*, quality of life; *RR*, relative risk.

Current evidence regarding CAM usage among patients with dermatomyositis remains sparse, consisting predominantly of case reports and small series with inherent reporting bias that emphasizes exceptionally positive or negative outcomes while underrepresentating of modest results, thereby hindering reliable conclusions. Additionally, the wide variety of CAM modalities with vastly different mechanisms and nature render direct comparison and synthesis challenging. Our categorical framing was intended to provide a preliminary structure, though we acknowledge that CAM outcomes often coexist on a spectrum rather than as discrete entities. Rather than drawing conclusions, our research maps the contour of an emerging area of interest and highlights key gaps where more robust research is needed and warning against potential harm that could result from unregulated usage of such readily available substances and interventions.

Further limitations include the inclusion of English full-text articles. Furthermore, 7 articles grouped DM with other myopathies, obscuring DM-specific findings. Finally, the absence of patient-reported outcomes in many studies limits our assessment of treatment impact.

This review reveals that current evidence is insufficient to draw meaningful conclusions about CAM effectiveness or safety in patients with DM. While some patterns emerge, the fundamental limitations in study design and reporting bias preclude clinical guidance. Well-designed controlled studies are needed to inform clinical practice.

## Conflicts of interest

AH is a consultant at CSL Behring and AstraZeneca, and an investigator for Priovant.
